# Supplementary material for: A Greater Adherence to the Mediterranean Diet Supplemented with Extra Virgin Olive Oil and Nuts During Pregnancy Is Associated with Improved Offspring Health at Six Years of Age
Source: Nutrients. 2025 May 19;17(10):1719. doi: 10.3390/nu17101719 (PMC12113803; doi:10.3390/nu17101719)
Supplement: Supplementary file 1 [file nutrients-17-01719-s001.zip › Supplementary Table S3.pdf]

Supplementary Table S3. Children's breastfeeding, cereal introduction daycare and vaccine calendar data at 6 years of age according to their mothers' glucose tolerance during pregnancy (GDM or NGT)

	**GDM**	**NGT**	***p***
Number	320	1488	
Gestation Age at delivery	39.4 ±1.7	39.5 ± 1.5	0.164
Age at 6 years follow-up (months)	70.3 ± 4.3	70.6 ± 4.4	0.139
Body Weight (Kg)	22.0 ± 4.3	22.2 ± 4.1	0.398
Z score	0.16 ± 1.17	0.014 ± 1.07	0.349
Height (cm)	117.4 ± 5.6	118.1 ± 5.4	0.145
Z score	0.07 ±1.23	0.19 ±1.18	0.274
BMI ($kg.m^{-2}$)	15.9 ± 2.2	16.0 ± 2.2	0.621
Z score	-0.13 ± 1.03	-0.07 ± 1.05	0.495
Breastfeeding	297 (93.0)	1358 (91.3)	0.318
Exclusive (months)	4.58 ±1.64	4.70 ±1.71	0.425
Mixed (months)	9.43 ± 7.73	9.22 ±7.21	0.743
Cereal Introduction (months)			
Gluten-free cereal	4.88 ± 0.88	4.95 ± 0.93	0.476
Gluten cereal	6.57 ± 1.23	6.50 ± 1.29	0.533
Daycare	222 (69.4)	994 (66.8)	0.411
Age (months)	12.3 ± 6.6	13.4 ± 6.7	0.087
Vaccinations Compulsory	317 (99.7)	1446 (97.2)	0.352
Vaccinations Recommended			
Meningitis	188 (58.7)	899 (60.4)	0.138
Rotavirus	212 (66.4)	1002 (67.3)	0.135
Others (A-hepatitis/influenza)	18 (5.7)	120 (8.1)	0.074
Covid-19	100 (31.2)	419 (28.2)	0.184
Covid-19 infection	84 (26.2)	447 (30.0)	0.047

Results expressed as mean ± SD or n (%). GDM, Gestational Diabetes Mellitius. NGT, Normal glucose tolerance. BMI, body mass index
