# Supplementary material for: A Greater Adherence to the Mediterranean Diet Supplemented with Extra Virgin Olive Oil and Nuts During Pregnancy Is Associated with Improved Offspring Health at Six Years of Age
Source: Nutrients. 2025 May 19;17(10):1719. doi: 10.3390/nu17101719 (PMC12113803; doi:10.3390/nu17101719)
Supplement: Supplementary file 1 [file nutrients-17-01719-s001.zip › Supplementary Table S4.pdf]

Supplementary Table S4. Children's breastfeeding, cereal introduction daycare and vaccine calendar data at 6 years of age according to mother's compliance to nutritional recommendations during pregnancy as modified Mediterranean Diet Adherence Screener Score (MEDAS) ≥6 vs. <6.

MEDAS	**≥6**	**<6**	***p***
Number (n)	695	1113	
Gestation Age at delivery	39.6 ± 1.5	39.5 ± 1.6	0.195
Age at 6 years follow-up (mo.)	70.0 ± 4.4	70.5 ± 4.3	0.057
Body Weight (Kg)	22.6 ± 4.2	22.6 ± 4.4	0.154
Z score	0.02 ± 1.03	0.08 ± 1.12	0.295
Height (cm)	117.8 ± 5.6	118.2 ± 5.5	0.236
Z score	0.13 ± 1.19	0.19 ± 1.15	0.365
BMI (kg.m^{-2})	15.9 ± 2.2	16.1 ± 2.5	0.352
Z score	-0.06 ± 1.03	-0.01 ± 1.12	0.411
Breastfeeding	647 (92.9)	1008 (90.6)	0.201
Exclusive (months)	4.95 ±1.66	4.64 ± 1.69	0.064
Mixed (months)	9.72 ± 7.30	9.07 ± 7.21	0.111
Cereal Introduction (months)			
Gluten-free cereal	4.91 ± 1.00	4.90 ± 1.00	0.903
Gluten cereal	6.59 ± 1.38	6.50 ± 1.23	0.215
Daycare	507 (73.0)	709 (63.7)	0.009
Age (months)	13.7 ± 6.8	13.5 ± 6.6	0.731
Vaccinations Compulsory	653 (94.0)	1110 (99.7)	0.459
Vaccinations Recommended			
Meningitis	451 (64.9)	636 (57.1)	0.015
Rotavirus	495 (71.2)	719 (64.6)	0.065
Others (A hepatitis/influenza)	52 (7.5)	86 (7.7)	0.320
COVID-19	220 (31.7)	299 (26.9)	0.017
COVID-19 infection	211 (30.4)	336 (30.2)	0.490

Results expressed as mean ± SD or n (%). BMI, body mass index; Mo, months
