# Supplementary material for: A Greater Adherence to the Mediterranean Diet Supplemented with Extra Virgin Olive Oil and Nuts During Pregnancy Is Associated with Improved Offspring Health at Six Years of Age
Source: Nutrients. 2025 May 19;17(10):1719. doi: 10.3390/nu17101719 (PMC12113803; doi:10.3390/nu17101719)
Supplement: Supplementary file 1 [file nutrients-17-01719-s001.zip › Supplementary Table S1 Characteristics of mothers gestation.pdf]

Supplementary Table S1. Characteristics of mothers gestation, delivery and newborn whose children have been analyzed at 6 years of age.

	CONTROL GROUP N=516	INTERVENTION GROUP N= 1292	SS
Gestational Age (weeks) at baseline	12.1 ± 0.6	12.0 ± 0.3	0.899
Pre-pregnancy Body Weight (kg)	62.2 ± 11.8	61.3 ± 10.7	0.070
Weight gain at:			
24-28 GW	7.5 ± 4.5	7.0 ± 4.3	0.033
36-38 GW	11.3 ± 6.9	11.9 ± 5.9	0.133
Systolic BP / Diastolic BP (mm Hg)			
12 GW	109 ± 11 / 67 ± 9	109 ± 10 / 67 ± 9	0.695 / 0.278
24 GW	106 ± 11 / 64 ± 9	106 ± 11 / 64 ± 8	0.485 / 0.802
36 GW	114 ± 13 / 71 ± 9	114 ± 13 / 72 ± 10	0.079 / 0.512
Fasting Blood Glucose (mg/dl)			
12 GW	80.8 ± 6.2	80.3 ± 6.1	0.084
24 GW	86.1 ± 7.0	84.8 ± 6.6	0.001
36 GW	78.4 ± 7.9	77.9 ± 9.6	0.010
GDM at 24-28 GW n (%)	112 (22.6)	208 (17.1)	0.006
Gestation Age at delivery	39.5 ± 1.5	39.5 ± 1.6	0.706
Cesarean Section n (%)	56 (15.3)	54 (16.0)	0.111
New born weight (grams)	3239 ± 458	3235 ± 487	0.887
Apgar 1/5 min. Values	8.8 ± 0.9 / 9.8 ± 0.6	8.8 ± 0.9 / 9.8 ± 0.6	0.697 / 0.776
Cord pH	7.27 ± 0.07	7.28 ± 0.11	0.723

Data are Mean ± SD or number (%) METS, Metabolic Syndrome. UNK, unknown. BMI, body mass index; GW, gestational weeks; GDM, Gestational Diabetes Mellitus; BP, blood pressure
