# Supplementary material for: A Greater Adherence to the Mediterranean Diet Supplemented with Extra Virgin Olive Oil and Nuts During Pregnancy Is Associated with Improved Offspring Health at Six Years of Age
Source: Nutrients. 2025 May 19;17(10):1719. doi: 10.3390/nu17101719 (PMC12113803; doi:10.3390/nu17101719)
Supplement: Supplementary file 1 [file nutrients-17-01719-s001.zip › Supplementary Table S2 Children.pdf]

Supplementary Table 2. Children's breastfeeding, cereal introduction and vaccine calendar data at 6 years of age according to whether their mothers belonged to the Intervention (IGC) or control group CGC).

	**CGC (n 516)**	**IGC (n 1292)**	***p***
Breastfeeding n	441 (93.0)	1214 (91.7)	0.204
Exclusive (months)	4.95 ± 1.61	4.67 ± 1.69	0.140
Mixed (months)	10.00 ± 7.43	9.31 ± 7.35	0.423
Cereal Introduction (months)			
Gluten-free cereal	4.83 ± 0.92	4.89 ± 0.95	0.359
Gluten cereal	6.62 ± 1.40	6.49 ± 1.29	0.136
Nursery	306 (64.3)	910 (69.1)	0.031
Age (months)	14.9 ± 6.4	13.2 ± 6.8	0.009
Vaccinations			
Compulsory	467 (98.7)	1296 (99.6)	0.494
Recommended			
Meningitis	255 (53.9)	832 (63.3)	0.009
Rotavirus	312 (66.0)	902 (68.6)	0.160
Others(A hepatitis, influenza)	11 (2.3)	127 (9.7)	0.001
COVID-19	179 (34.7)	340 (26.3)	0.001
COVID-19 infection	122 (23.6)	425 (32.9)	0.001

Results expressed as mean ± SD or n (%).
